# Comparison of Easy and Difficult Embryo Transfer Outcomes in *In Vitro* Fertilization Cycles

**Published:** 2013-03-03

**Authors:** Firouzeh Ghaffari, Kiandokht Kiani, Akram Bahmanabadi, Mohammadreza Akhoond

**Affiliations:** 1 Department of Endocrinology and Female Infertility at Reproductive Biomedicine Research Center, Royan Institute for Reproductive Biomedicine, ACECR, Tehran, Iran; 2 Department of Statistics, Mathematical Science and Computer Faculty, Shahid Chamran University, Ahwaz, Iran; 3 Department of Epidemiology and Reproductive Health at Reproductive Epidemiology Research Center, Royan Institute for Reproductive Biomedicine, ACECR, Tehran, Iran

**Keywords:** Embryo Transfer, Pregnancy Outcomes, Implantation

## Abstract

**Background:**

The aim of this study was to compare the effects of easy and difficult embryo transfers (ET) on implantation and pregnancy rates.

**Materials and Methods:**

In this prospective study, we analyzed the results of 706 ET procedures over a 12-month period. An easy ET was defined as a transfer that occurred without the use of force or other instrumentation. A difficult ET was defined as the use of force for catheter placement, and/or the use of additional instruments, and/or manipulation. Pregnancy rate was compared between patients with easy or difficult ETs.

**Results:**

There was a significantly higher implantation rate in the easy group (21.7%) compared to the difficult group (12.1%, p<0.05).The easy group had a higher pregnancy rate (38.1%) compared to patients who had difficult ETs (21.4%; p<0.05).

**Conclusion:**

Any uterine manipulation during ET adversely affects *in vitro* fertilization (IVF). Precaution should be taken to identify possibly difficult ET cases in advance.

## Introduction

The success of *in vitro* fertilization (IVF) depends on numerous factors such as embryo quality, infertility cause, endometrial receptivity, and the embryo transfer (ET) technique. An ET is the final step in an IVF cycle which, despite its simplicity, can adversely impact the IVF result. Many investigators have suggested that a meticulous ET technique is essential to IVF success (1-4). Unfortunately the performance of an ET is not as simple as it appears; poor ET technique accounts for 30% of all IVF failures (5).

Most ET are easy and do not require the use of force or manipulation. Different attempts have been suggested to prevent technically difficult ET, such as the performance of a dummy ET in order to ascertain the depth and direction of the uterus, (6,7) ultrasonography-guided ET for correct embryo placement; (8) and instructing the patient to have a full bladder for straightening the uterocervical angle (9). Despite these suggestions, there are a small group of patients for which ET is difficult and accomplished by the use of manipulation, which increases uterine contractions (10) and could affect embryo implantation (11).

Because a difficult ET has been shown to cause a significant reduction in pregnancy rate (12-14), therefore more attention should be focused on this simple, last step of the IVF cycle in order to improve the IVF outcome. The aim of this study is to compare the effects of easy and difficult ET on implantation and pregnancy rates.

## Materials and Methods

This prospective study was performed at Royan Institute for Reproductive Biomedicine over a 12- month period between May 2009 and May 2010. All eligible cases were included at study duration. We analyzed the results of 706 ET procedures. There were six specialists who performed all the ET procedures, each of them had more than four years of experience.

Inclusion criteria for participation in the study were: maternal age≤41 years, early follicular phase (day3) follicle stimulating hormone (FSH) ≤15 IU/L, and the presence of at least one grade A or B embryo on days 2-3 after oocyte retrieval.

Patients with a hydrosalpinx and abnormal uterine cavity, endometrial thickness <7mm at the time of hCG injection, or those who were candidates for blastocyst transfer, freeze-thaw embryo, oocyte donation, or surrogated cycles were excluded from this study.

This study was approved by the Ethics Committee of Royan Institute at the Reproductive Biomedicine Center. Written informed consent was obtained from all participants prior to study entry.

All patients received the standard long protocol as described elsewhere (15) and underwent ET 48-72 hours after oocyte retrieval. Embryo quality was assessed according to morphology, cleavage stage, and fragmentation rate (16). Progesterone supplementation (400 mg twice a day; Aburaihan Co., Tehran, Iran) began on the day of oocyte retrieval and continued until the day of the β-hCG assay. If the β-hCG result was positive, patients continued to receive progesterone until ten weeks of gestation.

All ET were performed using a soft ET catheter (Labotect GmbH, Göttingen, Germany) without ultrasound guidance. This catheter system has an outer sleeve and a soft open ended inner catheter. The patient was placed in the lithotomic position with an empty bladder)according to our center's protocol). A sterile bivalve speculum was placed in the vagina and the cervix exposed. Each ET was performed without anesthesia.

All patients underwent a mock ET or trial transfer performed 1-2 months before the IVF cycle with the intent to determine the uterine cavity depth and map the direction of the cervix and uterus.

After cleaning the cervix with a sterile swab soaked with saline followed by a dry swab, the outer sheet was passed gently through the external os until the tip passed the internal os. Then, an embryologist loaded the embryos into the transfer catheter after which they were deposited into the uterine cavity. The tip of the inner catheter was placed 6-6.5 cm from the external cervical os and embryos were placed 1-2 cm from the uterine fund us. After removal of the ET catheter, patients remained in the supine position for 20 minutes. We classified all ET procedures as easy or difficult. This study defined an easy ET procedure as a smooth procedure that occurred without the use of any force or other instrumentation, with no need to change the catheter. A difficult ET had at least one of the following problems: the placement of the outer sheet required force, use of a stylet, use of a tenaculum to grasp the cervix and/or manipulation with a hysterometer.

The primary endpoint was clinical pregnancy. We measured serum βhCG levels 14 days after ET and defined clinical pregnancy as the presence of a gestational sac with fetal heart activity at seven weeks of gestation. Pregnancy rate was calculated by dividing the number of clinical pregnancies detected by the number of patients (clinical pregnancies/ patient).

### Statistical analysis

Statistical analysis was performed using the Statistical Packages for Social Sciences (version 13.0; SPSS Inc., Chicago, IL, USA). The normality in the distribution of continuous variables was assessed using the Kolmogorov-Smirnov test. Inter-group differences of normally distributed continuous variables were assessed by parametric statistics (student’s t test), whereas non-parametric statistics (Mann-Whitney U test) were used if the data were not normally distributed. Significant differences were evaluated by the chi-square test to compare non-continuous variables. Data were expressed as mean ± standard deviation (SD) unless otherwise specified. Statistical significance was set at p<0.05.

## Results

From May 2009 to May 2010, we included 706 patients in this study. Of these, there were 81.4% (575) easy ET and 18.6% (131) graded as difficult. There were no significant differences between the two groups as to the baseline or cycle characteristics (Table 1).

Table 2 shows the distribution of the ET classification. Difficult ET was divided as follows: ET necessitating the use of force; astylet was used, and ET that used a tenaculum, or the combination of these methods.

Implantation was significantly higher in the easy group (21.7%) compared to the difficult group (12.1%; p<0.05; OR=2.014; 95% CI:1.404-2.890).

Additionally, the easy group also had a higher pregnancy rate (38.1%) compared with patients who had difficult ET (21.4%; p<0.05;OR= 2.263; 95% CI: 1.442-3.550).

In the easy ET group, 9(1.6%) out of 575 patients had early abortions, compared with one early abortion (0.8%) in the difficult ET group (p>0.05). Four patients in the easy ET group had ectopic pregnancies, but there were no ectopic pregnancies in patients who underwent difficult ET (Table 3).

**Table 1:** Comparison of baseline demographic and clinical characteristics of the two study groups during ICSI/ET cycles


	Group A (easy ET) (n= 575)	Group B (difficult ET) (n= 131)	P value

**Age (Y)**	31.05 ± 4.84	30.8 ± 5.28	0.590
**BMI (kg/m^2^)**	26.2 ± 4.89	26.3 ± 4.18	0.783
**Duration of infertility (Y)**	8.4 ± 4.94	8.4 ± 5.11	0.884
**Cause of infertility**			
**Male factor**	357 (62.1%)	82 (62.6%)	0.724
**Female factor**	99 (17.2%)	22 (16.8%)	
**Mixed factors**	84 (14.6%)	16 (12.2%)	
**Unexplained**	35 (6.1%)	11 (8.4%)	
**Type of infertility**			
**Primary**	547 (95.1%)	129 (98.5%)	0.087
**Secondary**	28 (4.9%)	2 (1.5%)	
**Duration of stimulation (D)**	10.2 ± 1.86	10.4 ± 2.27	0.224
**Day- 3 FSH (IU/L)**	6.6 ± 3.19	6.7 ± 3.26	0.860
**Day- 3 LH (IU/L)**	5.2 ± 3.81	5.2 ± 3.64	0.952
**Total number of oocytes retrieved**	10.1 ± 4.70	9.4 ± 4.45	0.131
**Total number of embryos formed (2PN)**	4.9 ± 2.98	4.6 ± 2.99	0.248
**Total number of good quality of embryos transferred (garde A, or B)**	2.12 ± 2.05	1.8± 2.03	0.103
**Endometrial thickness (mm)**	9.9 ± 1.76	9.7 ± 1.71	0.252


Values are as mean ± SD.

**Table 2:** Distribution of ET types


Types of ET	Number	Percent

**Easy**	575	81.4
**ET with force**	21	3
**ET with stylet**	17	2.4
**ET with tenaculum insertion**	61	8.6
**Combination of tenaculum and hysterometer**	4	0.6
**Combination of stylet, tenaculum, and hysterometer**	11	1.6
**Combination of stylet and tenaculum**	17	2.4
**Total**	706	100


**Table 3:** Cycle outcomes of 706 patients following difficult or easy ET


	Easy ET (n= 575)	Difficult ET (n= 131)	P value	Odds ratio (95% CI)

Clinical pregnancy rate*	219 (38.1)	28 (21.4)	<0.001	2.263 (1.442-3.550)
Implantation rate*	320/1474 (21.7)	38/314 (12.1)	<0.001	2.014 (1.404-2.890)
**Abortion rate**	9 (1.6)	1 (0.8)	0.483	2.067 (0.260-16.460)
**Ectopic pregnancy rate**	4 (0.7)	0 (0)	0.338	1.229 (1.187-1.274)


*;Statistically significant . Values in parentheses are percentages.

## Discussion

In this study, those who experienced difficult ET showed lower implantation and pregnancy rates. The impact of a difficult ET on the success of IVF cycles has remained a subject of debate in studies. Some studies have shown a negative correlation between difficult ET and pregnancy rate (17, 18) whereas other studies did not find any adverse effect in terms of pregnancy in difficult ET cycles (19, 20).These conflicting results can be attributed to the small number of patients and lack of consistent criteria for ET grading. In addition the patient selection has not been uniform among different studies. However, the contamination of the ET catheter by blood or mucus is common after a difficult ET as a result of trauma to endocervical canal. Under these circumstances the embryo may come back into the catheter during ET or blood may be drawn into the endometrial cavity, compromising embryo implantation. These factors may negatively impact pregnancy rates (21-23).

In the current study all patients underwent a mock ET or trial transfer performed 1-2 months before the IVF cycle; at that time the uterine cavity depth and direction of the cervix and uterus were mapped. Different treatments such as cervical dilatation by a Hegar dilator, (24) laminaria, (25) and hysteroscopic cervical canal shaving (26) havebeen proposed for improving cervical patency in patients with cervical stenosis (27). In this study after a difficult dummy transfer, if interring the dummy catheter was impossible (cervical stenosis), we performed cervical dilatation one month before stimulation. Despite the fact that performing mock ET before IVF cycles has been shown to improve pregnancy rates (7), some patients experienced a difficult ET with lower pregnancy rates. The mock ET could not completely improve their cervical stenosis or they experienced new cervical stenosis at the time of ET. In the present study, those patients who experienced difficult ET had a stylet placed inside the soft catheter. This technique converts a soft catheter into a stiff catheter. Several studies have investigated different kinds of catheters for ET and have demonstrated soft catheters to be superior in terms of pregnancy (3, 28, 29).The stiffer catheter may cause trauma and increase uterine contractions (30), however other studies have observed no difference (31). In women which acute cervico-uterine angulations that made ET difficult, the cervix was held by a tenaculum to introduce the catheter. Lesny et al. (10) have reported that tenaculum application to the cervix increases uterine contractions, which affect embryo implantation (11). In a small number of patients with cervical stenosis, cervical dilatation is the logical decision for overcoming this problem. In our center, we use a hysterometer for such cases. Visser et al. have reported no pregnancies in patients who had cervical dilatation performed two days before ET (14). In another study cervical dilatation on the oocyte retrieval day resulted in low numbers of pregnancies (32). However Tur-Kaspa et al. have reported that cervical dilatation during a difficult ET did not have any adverse effect regarding pregnancy rate (20). Possible endometrial trauma and uterine contraction may impair the implantation process. According to the lower pregnancy rate in the above procedures used for overcoming a difficult ET, other alternative methods such as trans-myometrial ET or cancelling the ET procedure may be considered. By obtaining patient agreement, embryos could be frozen and transferred in a subsequent cycle. Use of the versa point to refashion the cervical canal has been reported in a recent study. The authors believe this procedure is a useful technique to overcome unusually difficult ET (33). However, more trials with sufficient sample sizes are needed to confirm this result.

## Conclusion

ET should be smooth with easy passage of the transfer catheter. Since any uterine manipulation during ET adversely affects IVF results, therefore precaution should be taken to identify possibly difficult ET cases in advance. Additional studies regarding the numerous details of the ET technique appear to be essential.
